# Variations of Dietary Intake Across Migraine Phases in Adults with Episodic Migraine: A Prospective Observational Pilot Study

**DOI:** 10.1016/j.cdnut.2026.107668

**Published:** 2026-03-03

**Authors:** Maryann R Greenfield, Cara Frankenfeld, Anjali Pendala, Alexandra Ozmeral, Vyshnavi Pendala, Nora Baustian, Elizabeth K Seng, Jelena M Pavlovic, Raedeh Basiri, Margaret Slavin

**Affiliations:** 1 University of Maryland, Department of Nutrition and Food Science, College Park, MD, United States; 2 George Mason University, Department of Nutrition and Food Studies, Fairfax, VA, United States; 3 MaineHealth Institute for Research, Center for Interdisciplinary Population & Health Research, Scarborough, ME, United States; 4 Virginia Commonwealth University School of Medicine, Richmond, VA, United States; 5 Yeshiva University, Ferkauf Graduate School of Psychology, Bronx, NY, United States; 6 Albert Einstein College of Medicine, Saul R. Korey Department of Neurology, Bronx, NY, United States; 7 Montefiore Headache Center, Bronx, NY, United States

**Keywords:** migraine, headache, dietary recall, dietary quality, macronutrients

## Abstract

**Background:**

Migraine is a common and disabling neurological disorder with complex symptoms, which may include eating-related symptoms before, during, and after headache (HA) pain.

**Objectives:**

This study aimed to use a novel case-crossover design to assess dietary intake in temporal relation to migraine attacks.

**Methods:**

Data were collected from 25 people with migraine over a 28 d period, consisting of twice weekly dietary recalls combined with daily HA diaries assessing migraine symptoms. Micro- and macronutrient intakes were assessed, along with dietary quality. Generalized estimating equations assessed relationships between migraine attack phases and day-level dietary intake.

**Results:**

Participants included 23 females and 2 males, with a mean (M) age of 31 ± 12 y and with a mean migraine disability assessment score in the severe range at 28.6 ± 20.2. Vitamin D and choline had the lowest frequencies of participants meeting the Dietary Reference Intakes. During the prodrome phase, the Healthy Eating Index (HEI) component score for total protein foods [M: 4.6; 95% confidence interval (CI): 4.4, 4.9], calories from total protein (M: 19.6%; 95% CI: 17.1%, 22.0%), and animal protein (M: 13.4%; 95% CI: 10.8%, 16.1%) were significantly higher than on interictal days [(M: 4.1; 95% CI: 3.9, 4.4), (M: 15.7%; 95% CI: 14.5%, 16.9%), and (M: 9.6%; 95% CI: 8.4%, 10.8%), respectively]. On HA days with mild pain, total HEI scores [(M: 61.9; 95% CI: 53.8, 69.9), total vegetable HEI component (M: 4.4; 95% CI: 3.9, 5.0)], greens/beans component (M: 3.8; 95% CI: 2.7, 4.9) and fiber density (M: 15.6 g/1000 kcal; 95% CI: 12.8, 18.3 g/1000 kcal)], were higher than on HA days with severe pain [(M: 52.6; 95% CI: 47.5, 57.8), (M: 3.1; 95% CI: 2.5, 3.8), (M: 2.3; 95% CI: 1.4, 3.1), and (M: 11.1; 95% CI: 9.2, 13.0), respectively].

**Conclusions:**

A study design utilizing a novel combination of dietary recalls and HA diaries enables the observation of day-level differences in protein intake and dietary quality in relation to phase of the migraine attack and HA pain severity. The methodology is successful in consistently gathering detailed data that supports future designs, which represent diverse populations with migraine.

## Introduction

Migraine (MIG) disease is a common and disabling neurological disorder with complex interacting symptoms. It can be characterized by a headache (HA) that interferes with daily activity, along with nausea or vomiting, and/or sensitivity to light [1]. Formal diagnostic criteria also include the symptoms of moderate to severe pain intensity, aggravation by routine physical activity, sensitivity to sound, and a duration when left untreated of 4‒72 h [2]. Over 1 billion people globally have migraine, with prevalence peaking at over 30% for females in their late thirties [3]. Its disabling qualities combined with its prevalence lead to migraine as the second leading cause of disability globally, and among young females, it is the most common cause of disability [4].

**FIGURE 1:**
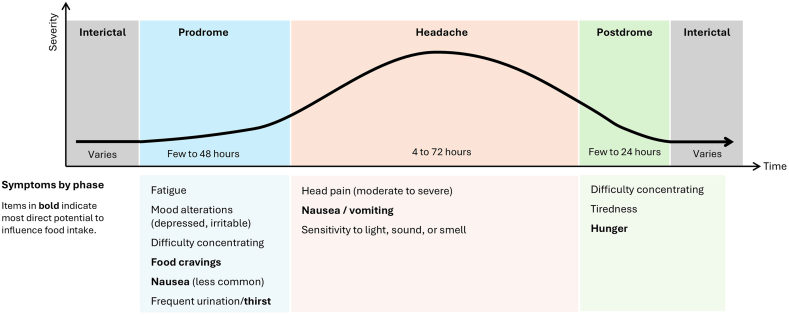
Migraine attack phase and select symptoms which may directly or indirectly influence food intake.

A migraine attack occurs in phases, each with distinct features. Prodrome (PRO) occurs in the hours to days prior to head pain onset; symptoms vary widely and can include sensitivity to light and sound and nausea, along with specific neurological signs (e.g., yawning, increased need to urinate), mood changes, neck pain, and food cravings [5]. For some people with migraine, the final component of the prodrome is the aura, in which people may experience visual, sensory, or language symptoms lasting ≤1 h immediately prior to head pain onset [2]. The migraine pain phase is characterized not only by severe pain, but also by associated symptoms including sensitivity to light, sound and smell, nausea, and sometimes vomiting. The 4‒24 h following head pain are considered the postdrome (POST), with typical symptoms of tiredness, cognitive difficulties, neck stiffness, and hunger [6]. The prodrome, aura, pain phase, and postdrome combined make up a migraine attack, whereas the time between attacks is known as the interictal (INT) phase and completes the migraine cycle. During the interictal phase, there may be anxiety or cognitive distress, concerns about making plans, and impairment in work or school and social life [7]. These phases and select symptoms that have the potential to impact diet are outlined in Figure 1.

Between nausea, food cravings, hunger, pain, and distress, there is ample opportunity for migraine to affect food choices via its symptoms, and this relationship is believed to be bidirectional, with potential for food choices to affect migraine outcomes [8]. Historically, if a food choice was perceived to precipitate a migraine attack, it was considered a dietary trigger for migraine [9]; however, recent evidence suggests an alternative explanation that prodrome symptoms could impact food choices in the hours to days prior to head pain onset [10]. Dietary patterns may also have a protective role in reducing migraine severity [11]. Dietary quality, as measured by the Healthy Eating Index (HEI), which assesses adherence to the Dietary Guidelines for Americans, has been found to differ in some groups of people with migraine compared with controls [[12], [13], [14]] and may influence migraine-related outcomes [[14], [15], [16], [17]]. People with migraine have been found to consume less of certain nutrients compared with Dietary Reference Intakes (DRIs) and be at higher risk for malnutrition [18]. Migraine characteristics have also been associated with body composition and biochemical test results [19]. Potential explanations for diet’s association with migraine explore both directions, including that 2 specific peptides, which are implicated in migraine physiology, have antiorexigenic effects that may influence appetite as they fluctuate during an attack [8], and the influence of pain on food choices and the desire to eat [20].

This bidirectional relationship makes the study of diet and migraine complex, with unique methodological challenges to be addressed. Dietary intake may vary throughout phases of the migraine attack, which requires an investigation that can distinguish these temporal differences while avoiding constant dietary assessments that can elicit fatigue and an associated loss of data quality [21]. To do this assessment, the dietary recall interview is recommended for prospective studies of diet associations [22], and inclusion of a multiple pass approach during the interview, which specifies several rounds of follow-up questions, improves accuracy [23] and has been validated for accurate assessment of calorie intake [24]. Case-crossover techniques have been noted for their ability to study multiple exposures in relation to migraine by recording on a sample of HA days and non-HA days, which reduces participant burden [25]; this design has been successful as the most common method to study migraine and air pollution [26]. These design features enable accurate identification of factors that precede migraine pain, which opens up opportunities for clinical interventions with preemptive therapies regardless of the directionality of any relationships [25].

The current objective is to demonstrate the novel application of prospective case-crossover methodology to study dietary intake in relation to migraine attacks. Intermittent dietary recall interviews are employed alongside HA diaries to assess and compare dietary intake across different phases of the migraine attack. In doing so, we describe nutritional intake and how it is temporally related to migraine attacks, comparing interictal days with days during, before, and after the pain phase of a migraine attack. We also explore HA pain intensity as an example of a migraine symptom for associations with dietary intake.

## Methods

### Participants

Adults with episodic migraine (4‒14 mo HA days) were recruited and observed longitudinally for 4 wk, including HA diaries every night and 24-h dietary recalls twice per week, with all data collected remotely. These steps are visualized in Supplementary Figure 1.

Eligible participants were ≥18 y old, with episodic migraine, with or without aura who were currently experiencing 4‒14 HA days per month, and were recruited and completed data collection between August 2022 and May 2023. Eligibility was not dependent on whether the migraine type was with or without aura. Established methods were used to accurately identify people with migraine prior to enrollment, consisting of an online screening using the American migraine Prevalence and Prevention [27] diagnostic module developed for the American migraine study, and the structured diagnostic interview for HA [28] approach using International Classification of Headache Disorders 3rd edition criteria [2] and administered via phone. Sources for recruitment included prior health research participants, university campus flyers, a HA clinic, and migraine-related public events. Excluded were those who were pregnant, breastfeeding, <6 mo postpartum, or planning a pregnancy within 3 mo; those with >10% change in body weight over the past 6 mo, chronic migraine, medication overuse HA, or a non-migraine HA as the predominant HA type; those with conditions that would interfere with data collection including significant psychiatric diagnoses, substance use, or cognitive impairment; and those with conditions or medications that would interfere with eating and appetite. The details of participants included/excluded are given in Supplementary Figure 2.

Ethical approval was obtained from the George Mason University Institutional Review Board (institutional review board number: 1866444), and all participants gave voluntary written consent prior to their enrollment.

### HA assessment

After enrollment, a baseline questionnaire collected information on demographics, health, and migraine history, along with height and weight, which were used to calculate BMI (in kg/m^2^). This questionnaire also included the validated migraine disability assessment, which scores the number of days in the past 3 mo in which various productive and leisure activities have been limited or affected by migraine attacks [29].

Migraine-related data were collected from HA diaries, which are a common instrument to study migraine that enables an accurate account of HA experiences and the factors surrounding them [30]. The HA diary link was sent as a text message 1 h before the participant’s reported typical bedtime, and connected to the REDCap (research electronic data capture) platform. It asked about daily occurrences that may be associated with migraine, such as illness, stress level, and sleep quality. For any HAs reported, a series of questions followed on the onset and offset time, pain intensity, medication use, and symptoms that would support identification of migraine attacks. Pain intensity ratings were categorized as mild for 1‒3, moderate for 4‒6, and severe for 7‒10.

### Migraine phases

Days on which head pain was reported were characterized using the start and end times to calculate the duration of the head pain. These times were then applied to categorize HA status by day, with a HA reported at any time between 00:00 and 23:59 indicating a HA day. If a HA presented with 2 of the 3 symptoms of nausea/vomiting, photophobia, or HA interference with daily activities (per 3-item ID Migraine screener [1]), or a MIG-specific acute medication (triptans, ditans, gepants, or ergotamine) was consumed, then the HA day was classified as a migraine day. Days with HAs without these symptoms or medications were classified and analyzed as “other headache” (OTHER) days. Classification then distinguished nonhead pain days, which were 1 d before migraine days as prodrome days, and similarly non-HA days, which were 1 d after migraine days as postdrome days. Finally, days without HA that remained unclassified after this were considered interictal.

### Dietary assessment

Diet was assessed by 24-h recall interviews with the multiple pass method, using the Nutrition Data System for Research (NDSR) 2022 software. Names, amounts, preparation, timing, and location were recorded for all foods and beverages consumed, and dietary supplement intake and its relation to typical intake were included. These interviews were conducted twice weekly for 4 wk, totaling 8 interviews per participant. Criteria of no >2 interviews on the same day of the week, no >2 consecutive days of interviews, and 2 or 3 weekend-day interviews were applied to support a varied and representative assessment of dietary intake. These were unannounced until the morning of the interview, assessing intake from 00:00 to 23:59 the previous day, and were completed via phone by trained interviewers. The principal investigator performed a quality check of the data, in accordance with NDSR recommendations, verifying that entries of foods and amounts matched what was reported.

Adequacy of micronutrients in the diet was assessed by percentage of the DRIs for each participant’s personalized life-stage group and gender [31,32]. The mean intake for each participant was used to calculate means and SDs for this sample overall. It was also categorized according to the individuals’ DRI as 0‒24%, 25‒49%, 50‒74%, 75‒99%, and ≥100% of the recommended intake. This was calculated for nutrients from foods alone and from foods and supplements combined.

Diet quality was assessed by day using the HEI-2020, which is designed to measure adherence to the Dietary Guidelines for Americans [33]. To present the adequacy of the diet quality, the percentage of days meeting the full score for each component was calculated. Foods were categorized by the Nova Classification System according to the 4 levels of processing [34], and used to calculate the percentage of calories consumed from ultraprocessed foods (level 4) per day. Two individuals independently coded each item, with any discrepancies adjudicated by the principal investigator.

### Statistical analysis

**FIGURE 2:**
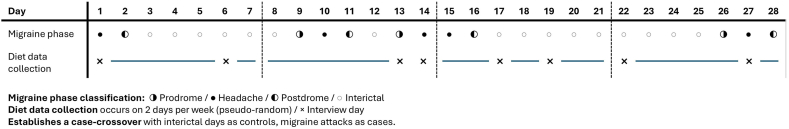
Example of how headache diary and dietary interview data were aligned and coded for analysis.

This analysis uses a case-crossover design, with each participant’s interictal days used as a control and all other days as cases, shown by example in Figure 2.

Summary statistics of personal characteristics were calculated on a per participant basis as means and SDs, or numbers and percentages for categorical variables. Comparisons between nutrients and HEI components with the migraine phase were made using generalized estimating equations (GEE), controlled for within-person correlations. Due to the high intraindividual nature of dietary intake, an independent correlation structure was used. Since individuals experienced migraine attacks naturally throughout the observation period, participants contributed dietary data on different numbers of migraine phase days. The GEE model was chosen for this purpose because it allows for different numbers of observations per participant, using all available data points for each participant to calculate marginal mean responses. Variables used in the GEE analysis were selected as energy-controlled values, including percentage of energy from macronutrients and HEI component scores. Energy itself was natural-log transformed for GEE comparisons. Means and SDs for each migraine attack phase were calculated using this model and were recorded prior to the natural-log transformation in the case of energy. The *P* values presented compare these to the reference group of interictal days. An adjusted model included stress level (0‒10) and sleep quality (1‒4) as covariates.

The same variable list and correlation structure were used to compare nutrients and HEI components with pain intensity, which includes only HA days. This HA characteristic is present to varying degrees on all HA days. Analyses were conducted in Stata, version 19.

## Results

### Participant characteristics

Participant characteristics are listed in Table 1. Demographics describing over 50% of the participants include those who were White, female, college graduates, and/or those with a household income over $75,000 per year. Migraine characteristics varied widely, but the migraine disability assessment score mean of 28.5 indicates a severe level of disability.

**TABLE 1:** Participant characteristics.

	Total mean [SD] or n (%)	
Baseline characteristics		
Gender		
Male∗	2	(8)
Female∗	23	(92)
Age (y)	31.4	[12.3]
BMI (kg/m^2^)	27.2	[7.1]
<18.5∗	1	(4)
18.5‒24.9∗	12	(48)
25 to <30∗	4	(16)
≥30∗	8	(32)
Race or ethnicity		
White∗	18	(72)
Asian∗	3	(12)
Other∗	4	(16)
Income (household $/y)		
<75,000∗	10	(40)
75,000 to <150,000∗	6	(24)
150,000+∗	9	(36)
MIDAS (at enrollment)	28.6	[20.2]
Years with migraine	15.7	[10.7]
Migraine with aura		
No∗	15	(60)
Yes∗	10	(40)
Headache characteristics observed during the study		
Participants’ daily stress level (0‒10 scale)	4.3	[1.5]
Participants’ daily sleep quality (1‒4 scale)	2.4	[0.5]
Participants’ headache frequency (days/28 d)	10.0	[4.3]
Participants’ headache frequency (attacks/28 d)	7.9	[3.4]

Abbreviation: MIDAS, migraine disability assessment.

∗n (%)

### Study compliance

With 26 participants who began the study, 1 was unenrolled due to noncompliance with diaries in the first week. Of the 25 remaining participants, 700 HA diaries and 200 dietary interviews were scheduled to occur. For the HA diaries of the 25 completers, 697 were completed, and 1 of the 3 missing diaries coincided with included dietary interview days. In total, 198 dietary interviews were completed. Out of these, 9 interviews were excluded from analysis due to acute illness that affected or was likely to affect dietary intake (e.g., COVID-19, flu, and food-borne illness), and 5 were excluded due to religious fasting, leaving 184 dietary interviews with representative dietary data.

### HA days observed

The total days with HA diary data, and its subset of days with dietary data, were classified, and the results are available in Table 2. In both the days with dietary data and the total days with HA diary data, interictal days were the most common. The percentages of days classified as each migraine phase were similar (within 2.5%) between days with dietary data and total days with HA diary data.

**TABLE 2:** Frequency of migraine attack phases observed in the analytical sample.

	Days with dietary and headache data *n* = 183^1^ *n* (%)		Days with headache diary data *n* = 697 *n* (%)	
**Interictal** (no headache on this day, and no migraine the day before or after)	71	(41.5)	284	(40.6)
**Prodrome** (1 d before migraine)	26	(14.2)	96	(13.9)
**Migraine headache** (headache on this day meets ID3 criteria for migraine)	56	(28.4)	181	(26.1)
**Other headache** (headache on this day does not meet ID3 criteria for migraine)	11	(8.2)	69	(9.8)
**Postdrome** (1 d after migraine)	19	(7.7)	67	(9.6)
**Total days observed/total planned**	198/200		697/700	

Abbreviation: ID3, 3-item ID Migraine screener.

1 Excludes 14 d dropped from dietary analysis due to illness or religious fasting, and 1 d without headache data.

### Micronutrient adequacy

**FIGURE 3:**
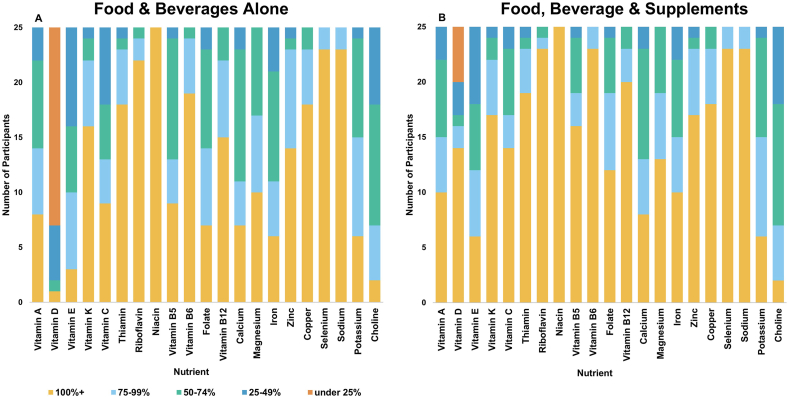
Frequency count of participants’ mean micronutrient intake compared to their Dietary Reference Intakes (DRI). (A) Mean intake from foods and beverages across all days for each participant compared to DRIs. (B) Mean intake from foods, beverages, and supplements across all days for each participant compared to DRIs. Where applicable, the recommended dietary allowance is used. All others are adequate intakes.

The adequacy of mean dietary intakes across all days recorded for each participant’s micronutrients as a percentage of their DRI, with and without supplements included, is graphed in Figure 3. To support this adequacy data, mean intakes of vitamins and minerals, both from foods and beverages alone and from their combination with supplements, are listed in Supplementary Table 1. Most participants consumed a mean of <25% of their DRI for vitamin D from foods and beverages alone, which is the only nutrient for which anyone consumed that little compared to the DRI. Supplements reduced this gap compared to the DRI, and vitamin D was the most common supplement consumed by participants. The most under-consumed nutrient with supplements accounted for was choline, with 2 of 25 participants consuming >100% of the DRI. Niacin was the only nutrient for which everyone consumed >100% of their DRI from foods and beverages alone.

### Dietary quality

The adequacy and moderation components, which make up the HEI, are presented as the criteria for a maximum score, along with the percentages of interviews that met these criteria and those for the minimum score, in Table 3. The highest number of interviews attaining the maximum score was found in total protein foods at 62%, followed by seafood and plant proteins. However, very few interviews were scored at 0 for total protein, whereas over one-third scored at 0 for seafood and plant proteins, leading to the high mean score for total protein foods at 4.1 of 5.0. On the lower end of the spectrum, whole grain adequacy had a mean of 4.0 of 10.0, with total fruit adequacy and sodium moderation just above that. The mean total score was 54.5 of 100.

**TABLE 3:** Healthy Eating Index-2020 scores attained.

Component	Maximum score criteria	% Scoring maximum	% Scoring minimum	Mean score (*n* = 184)
**Scored on a scale of 0‒5**				
Total fruits	≥0.8 cup equivalents per 1000 kcal	19	24	2.1
Whole fruits	≥0.4 cup equivalents per 1000 kcal	37	30	2.7
Total vegetables	≥1.1 cup equivalents per 1000 kcal	29	3	3.0
Greens and beans	≥0.2 cup equivalents per 1000 kcal	36	36	2.4
Total protein foods	≥2.5 oz equivalents per 1000 kcal	62	6	4.1
Seafood and plant proteins	≥0.8 oz equivalents per 1000 kcal	49	35	2.9
**Scored on a scale of 0‒10**				
Fatty acids	(PUFAs+MUFAs)/SFAs ≥2.5	23	14	5.2
Whole grains	≥1.5 oz equivalents per 1000 kcal	17	34	4.0
Dairy	≥1.3 cup equivalents per 1000 kcal	17	15	5.0
Refined grains	≤1.8 oz equivalents per 1000 kcal	23	18	5.4
Sodium	≤1.1 g per 1000 kcal	15	24	4.3
Added sugars	<6.5% of energy	38	4	8.0
Saturated fats	≤8% of energy	13	14	5.4
Total score	Sum of components = 100	0	0	54.5

### Case-crossover analysis of diet and migraine

**FIGURE 4:**
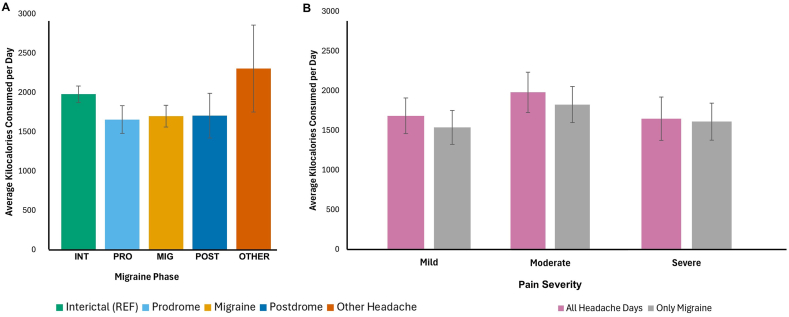
Mean daily energy intake on days categorized by (A) migraine phase and (B) pain severity. Bars represent mean values of kilocalories consumed per day for migraine phases (A) and by pain severity (B) as indicated. Error bars represent ±CI. For additional information, see Supplementary Tables 2 and 3. No comparisons were significant to *P* < 0.05 estimated by generalized estimating equations, controlled for within-person correlations, both adjusted and unadjusted for daily stress level and sleep quality, compared to interictal as the reference condition.CI: confidence interval; INT, interictal; MIG, migraine; OTHER, other headache; POST, postdrome; PRO, prodrome; REF, reference condition.

**FIGURE 5:**
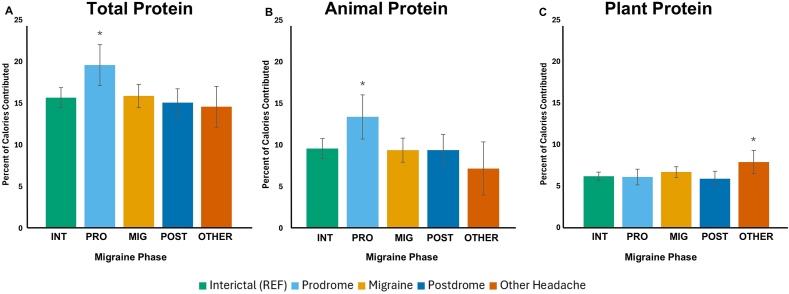
Mean percent of calories contributed by (A‒C) protein source on days categorized by migraine phase. (A) Mean percentage of calories from total protein for each migraine phase. (B) Mean percentage of calories from animal protein for each migraine phase. (C) Mean percentage of calories from plant protein for each migraine phase. Bars represent mean values of the percentage of calories contributed by protein source for migraine phases as indicated in the legend. Error bars represent ±CI. For additional information, see Supplemental Materials. ∗Values of *P* < 0.05 estimated by generalized estimating equations, controlled for within-person correlations, both adjusted and unadjusted for daily stress level and sleep quality, compared to interictal as the reference condition. CI: confidence interval; INT, interictal; MIG, migraine; OTHER, other headache; POST, postdrome; PRO, prodrome; REF, reference condition.

**FIGURE 6:**
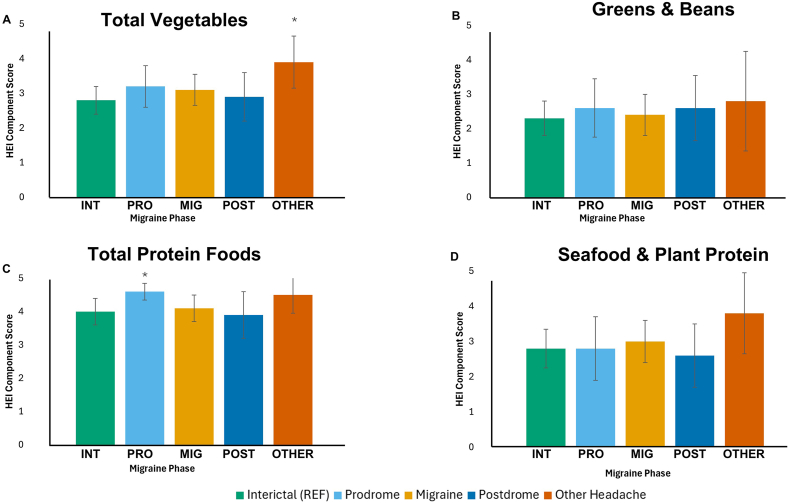
Mean Healthy Eating Index (HEI) (A and B) Vegetable and (C and D) protein components on days categorized by migraine phase. (A) Mean total vegetable component score for each migraine phase. (B) Mean greens and beans component score for each migraine phase. (C) Mean total protein food component score for each migraine phase. (D) Mean seafood and plant protein component score for each migraine phase. Bars represent mean values of HEI component scores for migraine phases as indicated in the legend. Error bars represent ±CI. For additional information, see Supplementary Materials. ∗Values of *P* < 0.05 estimated by generalized estimating equations, controlled for within-person correlations, both adjusted and unadjusted for daily stress level and sleep quality, compared to interictal as the reference condition. CI: confidence interval; INT, interictal; MIG, migraine; OTHER, other headache; POST, postdrome; PRO, prodrome; REF, reference condition.

**FIGURE 7:**
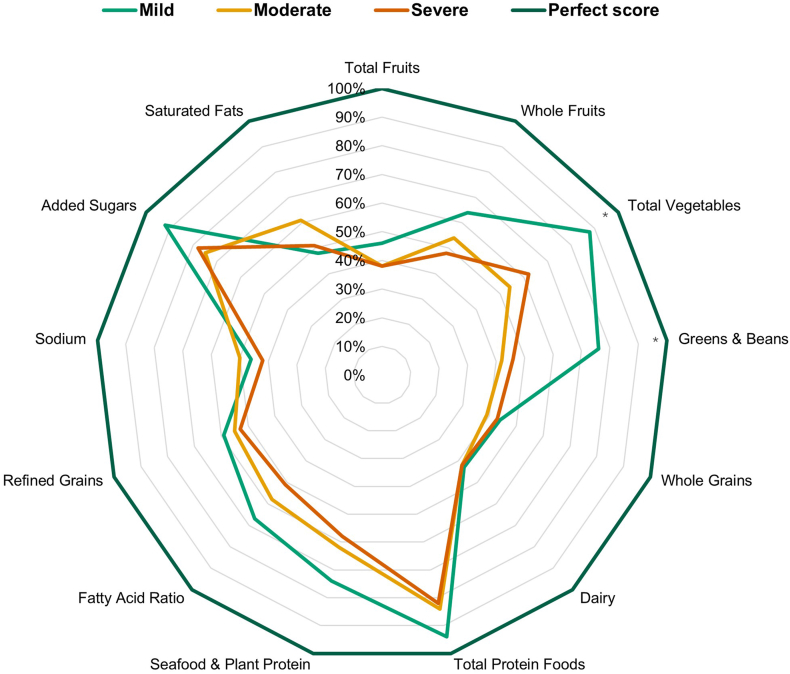
Mean daily percent of maximum scores for Healthy Eating Index (HEI) components on days with any headache, categorized by pain severity. Points represent mean values of HEI component scores by pain intensity from all headaches as indicated in the legend. Total score means (95% confidence intervals) were 61.9 (53.8, 69.9) for mild pain, 54.6 (49.8, 59.5) for moderate pain, and 52.6 (47.5, 57.8) for severe pain. For additional information, see Supplementary Materials. ∗Values of *P* < 0.05 by generalized estimating equations, controlled for within-person correlations, compared with severe pain as the reference condition.

Highlights from the GEE comparison between nutrients and dietary quality by migraine phase and by HA pain intensity are presented in Figures 4‒7, with full results in Supplementary Tables 2 and 3. Migraine phases of prodrome, migraine HA, postdrome, and other headache days are compared with interictal days as the reference, whereas pain intensities of mild and severe are compared to a severe pain reference.

Energy intake had the highest mean on HA days that did not meet migraine criteria, at 2304 kcal, and lower means were observed throughout the migraine attack phases at 1656 prodrome, 1699 migraine HA, and 1705 postdrome days, although no significant differences were found for this variable (Figure 4). Participants consumed significantly more protein on prodrome days than interictal days, as percentage calories from protein, percentage calories from animal protein (Figure 5), and the HEI component score for total protein foods (Figure 6). On days classified as other headache, percentage calories from plant protein (Figure 5) and the HEI component score for total vegetables (Figure 6) were higher than on interictal days. All of these results remained significant in both the adjusted and unadjusted models.

When analyzing pain intensity on both migraine and other headache days, total HEI scores on mild pain days had a mean of 61.9, and were significantly higher than severe pain days, which had a mean over 9 points lower (Figure 7). The same direction of total HEI scores was observed for days with only migraine pain, although not significant. Directionally, on mild head pain days, 12 out of 13 components scored higher than on severe head pain days, indicating an overall broader pattern of dietary quality, not just a single food type. Similarly, fiber density (Supplementary Table 3), total vegetable and greens and beans scores (Figure 7) were also significantly higher on mild pain days in both versions of this analysis. When evaluating only migraine HA days, calories from PUFA, ω-3, and ω-6 fatty acids were all higher on mild pain days compared with severe pain days. Energy intake trended higher than either mild or severe pain by over 200 kcal on moderate pain days, although no significant differences were found for this variable.

## Discussion

The present study observes patterns of dietary intake in 25 people with migraine, using ≤8 dietary recalls per participant. By aligning these with daily HA diaries, the intraindividual daily variations in diet that coincide with phases of migraine attacks, which are classified as prodrome, migraine HA, postdrome, interictal, and other headache days, are effectively captured. Since every day with a HA included a pain intensity rating, this study was also successful in associating HA pain severity with dietary intake. The rich nature of the dietary recall is precise in assessing nutrients and dietary quality, and the criteria for scheduling dietary interview days ensure that dietary data well represent the participants’ current diets. The subset of days for which dietary data was captured was also representative of the breakdown of all types of days captured in the HA diary in this sample (Table 2).

In the present analysis, all macronutrients and HEI components have been expressed as a ratio of kilocalories (kcal). The overall mean caloric intake of 1842 (1787 ± 652 for females only) is similar to the 1800 and 1808 for United States females with and without migraine reported elsewhere [13]. When evaluating results that are based on caloric ratios, it is worth noting that nominal differences in mean intake were observed (although not directly compared because neither was the reference group) of ∼600 fewer calories on migraine days than on other headache days, and calories peaking at 300 calories higher on moderate pain days than on mild pain days.

The association observed between mild pain intensity and increased dietary quality, as HEI scores, seems to be due to an especially high score on mild pain days, rather than an especially low score on severe pain days. The mean overall HEI score for this sample of 54.5 is closer to the mean for severe (52.6) than it is to mild (61.9), and is similar to the mean of 55.3 that has been observed in Americans ages 19‒59 [33]. It is worth noting that when diagnosing migraine, HAs of moderate to severe intensity are included as a criterion [2], so mild HA pain for a person with migraine may represent a different presentation of symptoms, or a day when acute medications were consumed to prevent pain from escalating. The 9-point HEI difference between mild and severe pain is well above the 5‒6 points recommended to consider clinical significance [35], and is similar in magnitude to differences that have been associated with migraine-related outcomes across individuals [14,17]. Although not a component of dietary quality, fiber density approaches values 50% higher for mild pain days compared with moderate pain days, and the mild pain day mean of over 15 g/1000 kcal puts it over the 14 g/1000 kcal recommendation in the Dietary Guidelines for Americans [36]. This was accompanied by total vegetable scores, which are often a source of fiber that were ∼2 points higher on a 5-point scale on mild pain days than on moderate pain days. In 1 cross-sectional study, macronutrient intake along with fiber was all correlated with migraine pain intensity on a per-person basis [19], which the current study supports in terms of fiber on days with higher pain intensity.

The adequacy of participants’ diets broadly, although not exactly, follows patterns of dietary consumption by Americans [18,37], including the nutrients consumed the least relative to the DRI of vitamin D [38] and choline [39]. Although significant differences in dietary consumption of micronutrients have been previously observed between people with migraine and people without [18], the current data did not indicate nutritional deficiencies specific to the eating patterns of people with migraine. Although differences in sugar or ultraprocessed foods could be expected based on the literature that people with migraine eat differently, these were not evident in the present analyses. The most notable micronutrient differences in the present study were observed in means for supplement consumption, including much higher riboflavin and magnesium supplementation compared to that observed in American females more broadly [37], which are commonly recommended for people with migraine due to their evidence for prophylaxis [40,41]. Although most participants are meeting their riboflavin needs, about half are not meeting their recommended intake of magnesium. This is despite that 9 of 25 participants reported taking a magnesium dietary supplement at least once, and 5 of those reported it in every interview, which supports high rates of inconsistent supplement usage (Supplementary Table 1).

Differences in dietary patterns across migraine phases revealed in this analysis include a higher level of protein during prodrome days and a higher level of vegetables during other headache days. The protein during prodrome days may be related to multiple biological underpinnings, including appetite changes through migraine attacks [8], and the perception of increased satiety from protein [42], or various possibilities for amino acid metabolism to associate with migraine attacks [43]. There are also analogous variations reported in protein, including animal protein, across days of the menstrual cycle [44]. Meanwhile, the higher intake of total vegetables and calories from plant protein on other headache days may relate to the HA symptoms reported, which, by definition, did not meet 2 of the 3 criteria to be classified as migraine. In this dataset, no days classified as other headache met the criterion of nausea as a symptom; only 1 had photophobia, whereas most did experience interference with daily activities. There was not enough data to assess the impact of nausea or photophobia on dietary quality directly, but it is possible that the increase in vegetables may be related to the lack of nausea. This is worth investigating in a larger study, and it would be supported by prior research that severe nausea was associated with reduced vegetable intake in pregnant females [45], and an inverse association was found between a dietary pattern high in fruits and vegetables and nausea in cancer patients [46].

The sample size for this pilot study limited statistical power. Without a sampling method that represents the broad population with migraine, there is the potential for any generalized recommendations to people with migraine to be speculative rather than evidence-based. However, this provides a foundation and a framework for a larger study. These temporal relationships based on day-level classifications are imprecise in assigning migraine phases, which may be less than a full day long, but any identification of time relative to migraine attack onset is useful in presenting opportunities for preemption [25], and the day-level classifications enable comparisons with the full day of 24-h recall data. The number of dietary recall interviews and HA diaries place burden on the participant to complete, but the high rate of compliance in this study suggests that this methodological limitation was well managed.

Among the many symptoms of migraine, some individuals experience cognitive impairment at different phases of an attack. This study used the dietary recall interview method, which relies on memory of foods eaten the previous day, thus there is the potential for these symptoms to impact reporting accuracy to varying degrees during migraine phases. Cognitive difficulty is most likely to occur during the HA phase, but may also affect the prodrome and postdrome [47]. Nonetheless, several factors make it difficult to predict the direction and extent of potential bias on the present results, including: the duration of HA phases is variable, the start/stop of each phase cannot be ascertained precisely, and the use of acute medications may improve cognitive deficits. The NDSR software utilized for dietary interviews includes a multipass interview approach to aid recall, and specifically probes for a wide range of foods that are commonly forgotten, including beverages, sweets, snacks, fruits, vegetables, cheese, and bread [48]. To mitigate the potential for bias in future studies, larger sample sizes would be needed. Assessment of differential exposure measurement error could enable adjustment for bias in exposure (dietary) measurements [49]. Alternate dietary assessment methods may also be considered, although methods that do not rely on memory (food diaries and photo diaries) may introduce a different kind of reporting bias, which would warrant further consideration.

Strengths of this research include the rigorous screening for people with migraine using validated tools [28] and the quantity of rich dietary data to examine dietary intake. The details collected allow for the application of a range of measurements of contemporary interest, which may be studied further, including HEI and ultraprocessed foods. In addition to using the gold standard of the structured diagnostic interview for HA to correctly assign the HA diagnosis in a non-clinical setting, the assignment of specific attacks as migraine or non-migraine HA was rigorously adhered to as well, further differentiating this study from prior studies discussed above. The electronic HA diary, which was used to report attacks, included a timestamp, which preempts the potential recall bias from retrospective recording that a paper diary would allow [25]. The strict screening process may also have contributed to the high adherence to the study protocol, in addition to the frequent contact with the study team via interviews. Dietary data were collected using what is currently considered the practical gold standard for nutritional assessments, the multipass 24-h recall interview. Using 8 dietary recalls, on a variety of days, allows for greater confidence that dietary intake is representative of typical intake and avoids the tendency toward fatigue in repeated daily assessments. It is only by having such a large number of recalls that there is a high chance of observing dietary intake on days with and without a migraine attack, enabling the novel use of a case-crossover analytical approach, which controls for within-person variations. To our knowledge, this is a novel application of these assessments that enables observations that have not been made before. With this approach, the present study was able to compare nutrient intake across days in temporal relation to migraine attack onset.

Future research directions would clarify relationships that may form distinct patterns based on migraine characteristics, which vary between individuals and attacks. Although analyses based on day-level classifications of migraine attack phases and pain intensity revealed significant associations, a larger dataset would be needed to subdivide based on specific symptoms, prodromal (premonitory) symptoms, menstrual phase, or specific hours in relation to attack onset. Days with HA that did not show symptoms of migraine were limited in number, and not directly compared with migraine days, but did appear different and could show important distinctions in a study designed to investigate this. It was also interesting that days with mild pain had such high HEI scores, higher than any migraine phase as a whole, and more data is needed to confirm this observation.

In conclusion, use of a prospective electronic HA diary in conjunction with randomized dietary recalls is a successful design that observes day-level differences in dietary intake and quality in relation to phase of the migraine attack and HA pain severity. The high rate of compliance shows that the methodology can consistently gather the detailed data informing the relationship between migraine and its symptoms on dietary factors, and supports its use in future designs that represent diverse populations with migraine. Future research could expand results to investigate particular symptoms of migraine with respect to potential effects on food intake and dietary quality.

## Author contributions

The authors’ responsibilities were as follows–MS, CF, EKS: designed the research; MS, AP, MRG, AO, VP, EKS, JMP: conducted the research; MRG, CF, MS: analyzed the data; MRG, MS, RB: wrote the manuscript; MS: had primary responsibility for the final content; and all authors: read and approved the final manuscript.

## Data availability

Data described in the manuscript and analytic code will be made available upon request after publication upon reasonable request, approval by the study team, and a data transfer agreement.

## Funding

This study was funded by the George Mason University College of Public Health and protocol approved by the George Mason University Institutional Review Board (institutional review board number: 1866444). This source had no involvement or restrictions regarding publication.

## Conflict of interest

MS reports receiving research funding from the United States Department of Agriculture, and travel reimbursement from the American Headache Society, Southern Headache Society and Headache Cooperative of the Pacific. ES reports receiving research funding from the National Institutes of Health and the American Heart Association, and has consulted, advised or received travel funding from GlaxoSmithKline Inc, Eli Lilly and Company, Click Therapeutics Inc, and AbbVie Inc. JP reports receiving travel reimbursement from the American Headache Society and International Headache Society. CF reports receiving research fundign from the National Institutes of Health and the United States Department of Agriculture. All other authors declare they have no known competing financial interests or personal relationship that could have appeared to influence the work reported in this paper.

## References

[1] Lipton R.B., Dodick D., Sadovsky R., Kolodner K., Endicott J., Hettiarachchi J. (2003). A self-administered screener for migraine in primary care: the ID Migraine validation study. Neurology.

[2] Headache Classification Committee of the International Headache Society (IHS) (2018).

[3] GBD 2016 Headache Collaborators (2018). Global, regional, and national burden of migraine and tension-type headache, 1990-2016: a systematic analysis for the Global Burden of Disease Study 2016. Lancet Neurol.

[4] Steiner T.J., Stovner L.J., Jensen R., Uluduz D., Katsarava Z. (2020). Lifting The Burden: the Global Campaign against Headache. Migraine remains second among the world’s causes of disability, and first among young women: findings from GBD2019. J. Headache Pain.

[5] Hasirci Bayir B.R., Gursoy G., Pul M.F. (2022). Frequency of prodromal symptoms in patients suffering from migraines with aura. Haseki.

[6] Karsan N., Peréz-Rodríguez A., Nagaraj K., Bose P.R., Goadsby P.J. (2021). The migraine postdrome: spontaneous and triggered phenotypes. Cephalalgia.

[7] Buse D.C., Rupnow M.F., Lipton R.B. (2009). Assessing and managing all aspects of migraine: migraine attacks, migraine-related functional impairment, common comorbidities, and quality of life. Mayo Clin. Proc..

[8] Martins-Oliveira M., Tavares I., Goadsby P.J. (2021). Was it something I ate? Understanding the bidirectional interaction of migraine and appetite neural circuits. Brain Res.

[9] Hindiyeh N.A., Zhang N., Farrar M., Banerjee P., Lombard L., Aurora S.K. (2020). The role of diet and nutrition in migraine triggers and treatment: A systematic literature review. Headache.

[10] Karsan N., Bose P., Newman J., Goadsby P.J. (2021). Are some patient-perceived migraine triggers simply early manifestations of the attack?. J. Neurol..

[11] Tu Y.H., Chang C.M., Yang C.C., Tsai I.J., Chou Y.C., Yang C.P. (2025). Dietary patterns and migraine: insights and impact. Nutrients.

[12] Martins L.B., Braga Tibães J.R., dos Santos Rodrigues A.M., Hassanzadeh Keshteli A., Karam Vono C., Borges J.B.E (2022). The quality and inflammatory index of the diet of patients with migraine. Nutr. Neurosci..

[13] Evans E.W., Lipton R.B., Peterlin B.L., Raynor H.A., Thomas J.G., O’Leary K.C. (2015). Dietary intake patterns and diet quality in a nationally representative sample of women with and without severe headache or migraine. Headache.

[14] Fotros D., Noormohammadi M., Togha M., Ghorbani Z., Hekmatdoost A., Rafiee P. (2024). Healthy eating index 2015 might be associated with migraine headaches: results from a Case–Control study. Food Sci. Nutr..

[15] Hajjarzadeh S., Nikniaz Z., Shalilahmadi D., Mahdavi R., Behrouz M. (2019). Comparison of diet quality between women with chronic and episodic migraine. Headache.

[16] Feyzpour M., Sedgi F.M., Baghdadi G., Mohammadifard R., Rahimlou M. (2024). Investigating the relationship between diet quality, lifestyle and healthy eating index with severity and migraine attacks: a cross-sectional study. Front Nutr.

[17] Balali A., Karimi E., Kazemi M., Hadi A., Askari G., Khorvash F. (2024). Associations between diet quality and migraine headaches: a cross-sectional study. Nutr. Neurosci..

[18] Peng C., Gao L., Wu K., Jiang X., Chen X., Li C. (2023). Association between the prognostic nutritional index and severe headache or migraine: a population-based study. Nutr. Neurosci..

[19] Bakırhan H., Yıldıran H., Uyar Cankay T.U. (2023). Dietary patterns and migraine: are dietary intake and biochemical parameters associated with migraine characteristics? Nutr. Food Sci.

[20] Darbor K.E., Lench H.C., Carter-Sowell A.R. (2016). Do people eat the pain away? The effects of acute physical pain on subsequent consumption of sweet-tasting food. PLOS One.

[21] Rebro S.M., Patterson R.E., Kristal A.R., Cheney C.L. (1998). The effect of keeping food records on eating patterns. J. Am. Diet Assoc..

[22] Thompson F.E., Kirkpatrick S.I., Subar A.F., Reedy J., Schap T.E., Wilson M.M. (2015). The National Cancer Institute’s dietary assessment primer: a resource for diet research. J. Acad. Nutr. Diet..

[23] Moshfegh A.J., Rhodes D.G., Baer D.J., Murayi T., Clemens J.C., Rumpler W.V. (2008). The US Department of Agriculture Automated Multiple-Pass Method reduces bias in the collection of energy intakes. Am. J. Clin. Nutr..

[24] Blanton C.A., Moshfegh A.J., Baer D.J., Kretsch M.J. (2006). The USDA automated multiple-pass method accurately estimates group total energy and nutrient Intake. J. Nutr..

[25] Lipton R.B., Pavlovic J.M., Haut S.R., Grosberg B.M., Buse D.C. (2014). Methodological issues in studying trigger factors and premonitory features of migraine. Headache.

[26] Portt A.E., Orchard C., Chen H., Ge E., Lay C., Smith P.M. (2023). Migraine and air pollution: A systematic review. Headache.

[27] Lipton R.B., Diamond S., Reed M., Diamond M.L., Stewart W.F. (2001). Migraine diagnosis and treatment: results from the American migraine Study II. Headache.

[28] Andrew M.E., Penzien D.B., Rains J.C., Knowlton G.E., McAnulty R.D. (1992). Development of a computer application for headache diagnosis: the headache diagnostic system. Int. J. Biomed. Comput..

[29] Stewart W.F., Lipton R.B., Kolodner K.B., Sawyer J., Lee C., Liberman J.N. (2000). Validity of the Migraine Disability Assessment (MIDAS) score in comparison to a diary-based measure in a population sample of migraine sufferers. Pain.

[30] Moloney M.F., Aycock D.M., Cotsonis G.A., Myerburg S., Farino C., Lentz M. (2009). An Internet-based migraine headache diary: issues in Internet-based research. Headache.

[31] Taylor C.L., Yaktine A.L., Ross A.C. (2011). Dietary Reference Intakes for Calcium and Vitamin D.

[32] National Academies of Sciences (2019).

[33] Shams-White M.M., Pannucci T.E., Lerman J.L., Herrick K.A., Zimmer M., Meyers Mathieu K.M. (2023). Healthy eating Index-2020: review and update process to reflect the dietary guidelines for Americans, 2020-2025. J. Acad. Nutr. Diet..

[34] Sneed N.M., Ukwuani S., Sommer E.C., Samuels L.R., Truesdale K.P., Matheson D. (2023). Reliability and validity of assigning ultraprocessed food categories to 24-h dietary recall data. Am. J. Clin. Nutr..

[35] Kirkpatrick S.I., Reedy J., Krebs-Smith S.M., Pannucci T.E., Subar A.F., Wilson M.M. (2018). Applications of the healthy eating index for surveillance, epidemiology, and intervention research: considerations and caveats. J. Acad. Nutr. Diet..

[36] U.S. Department of Agriculture and U.S. Department of Health and Human Services (2020).

[37] Freedman M.R., Fulgoni V.L., Lieberman H.R. (2024). Temporal changes in micronutrient intake among United States Adults, NHANES 2003 through 2018: A cross-sectional study. Am. J. Clin. Nutr..

[38] Moore C., Murphy M.M., Keast D.R., Holick M.F. (2004). Vitamin D intake in the United States. J. Am. Diet Assoc..

[39] Zuk E., Nikrandt G., Chmurzynska A. (2024). Dietary choline intake in European and non-European populations: current status and future trends-a narrative review. Nutr. J..

[40] Slavin M., Li H., Khatri M., Frankenfeld C. (2021). Dietary magnesium and migraine in adults: A cross-sectional analysis of the National Health and Nutrition Examination survey 2001-2004. Headache.

[41] Li H., Krall J.R., Frankenfeld C., Slavin M. (2022). Nutritional intake of riboflavin (vitamin B2) and migraine: a cross-sectional analysis of the National Health and Nutrition Examination Survey (NHANES) 2001-2004. Nutr. Neurosci.

[42] Veldhorst M., Smeets A., Soenen S., Hochstenbach-Waelen A., Hursel R., Diepvens K. (2008). Protein-induced satiety: effects and mechanisms of different proteins. Physiol. Behav..

[43] Biringer R.G. (2022). Migraine signaling pathways: amino acid metabolites that regulate migraine and predispose migraineurs to headache. Mol. Cell Biochem..

[44] Gorczyca A.M., Sjaarda L.A., Mitchell E.M., Perkins N.J., Schliep K.C., Wactawski-Wende J. (2016). Changes in macronutrient, micronutrient, and food group intakes throughout the menstrual cycle in healthy, premenopausal women. Eur. J. Nutr..

[45] Crozier S.R., Inskip H.M., Godfrey K.M., Cooper C., Robinson S.M., SWS Study Group (2017). Nausea and vomiting in early pregnancy: effects on food intake and diet quality. Matern. Child Nutr..

[46] Lei Y.Y., Ho SC S.C., Kwok C., Cheng A., Cheung K.L., Lee R. (2022). Association of high adherence to vegetables and fruits dietary pattern with quality of life among Chinese women with early-stage breast cancer. Qual. Life Res..

[47] Fernandes C., Dapkute A., Watson E., Kazaishvili I., Chądzyński P., Varanda S. (2024). Migraine and cognitive dysfunction: a narrative review. J. Headache Pain..

[48] Austin M., Brelje K., Harnack L., Jasthi B., Peasley J.L., Pettit J. (2022). Nutrition Coordinating Center. University of Minnesota, Nutrition Data Systems for Reasearch User Manual, Regents of the.

[49] White E. (2003). Design and interpretation of studies of differential exposure measurement error. Am. J. Epidemiol..

