# Supplementary material for: Variations of Dietary Intake Across Migraine Phases in Adults with Episodic Migraine: A Prospective Observational Pilot Study
Source: Curr Dev Nutr. 2026 Mar 3;10(4):107668. doi: 10.1016/j.cdnut.2026.107668 (PMC13068852; doi:10.1016/j.cdnut.2026.107668)

**Supplementary Materials**

**Table of Contents**

**Supplementary Table 1.** Mean Intake of Micronutrients of Adults with Episodic Migraine Over All Dietary Recalls [2](https://docs.google.com/document/d/1y0ZYSojAt5Rc8TERHdfpknyzG3cfPftb/edit#heading=h.ro2csnm94ah0)

**Supplementary Table 2.** Nutrient and Dietary Quality Comparison with Migraine Attack Phase3

**Supplementary Table 3.** Nutrient and Dietary Quality Comparison with Pain Intensity4

**Supplementary Figure 1**: Study Design Flow Diagram 5

**Supplementary Figure 2**: Participants Included and Excluded6

**Supplementary Table 1.** Mean Intake of Micronutrients of Adults with Episodic Migraine Over All Dietary Recalls

| **Nutrient**  (unit of measure) | **Intake from**  **foods only**  Mean (SD) | **Intake from**  **foods & supplements**  Mean (SD) | **Participants taking supplements**  (in all interviews/ in any interview) |
| --- | --- | --- | --- |
| Vitamin A (mcg RAE) | 635.8 (450.1) | 683.8(494.4) | - |
| Vitamin D (mcg) | 4.6 (9.3) | 43.0 (70.7) | 5/13 |
| Vitamin E (mg TE) | 10.1 (5.6) | 11.7 (7.1) | - |
| Vitamin K (mcg) | 130.9 (180.5) | 142.6 (184.4) | - |
| Vitamin C (mg) | 67.3 (66.8) | 134.0 (184.8) | 1/4 |
| Thiamin (mg) | 1.7 (1.6) | 6.9 (19.5) | - |
| Riboflavin (mg) | 1.8 (1.0) | 32.5 (105.2) | 0/2 |
| Niacin (mg NE) | 36.6 (17.6) | 42.9 (21.9) | - |
| Vitamin B5 (mg) | 4.7 (2.3) | 8.9 (26.1) | - |
| Vitamin B6 (mg) | 1.7 (1.0) | 3.2 (6.2) | - |
| Folate (mcg DFE) | 340.0 (184.6) | 505.2 (479.9) | - |
| Vitamin B12 (mcg) | 3.6 (5.2) | 126.5 (730.5) | 1/1 |
| Calcium (mg) | 873.0 (465.9) | 897.0 (474.2) | 0/1 |
| Magnesium (mg) | 298.7 (116.9) | 394.0 (235.5) | 5/9 |
| Iron (mg) | 13.3 (7.0) | 21.4 (20.9) | 2/4 |
| Zinc (mg) | 9.0 (4.6) | 13.9 (15.4) | 1/4 |
| Copper (mg) | 1.3 (0.6) | 1.3 (0.6) | - |
| Selenium (mcg) | 105.0 (59.5) | 106.5 (61.7) | - |
| Sodium (mg) | 3,077.5 (1,599.1) | 3,093.5 (1,602.9) | - |
| Potassium (mg) | 2,323.7 (1,032.8) | 2,330.2 (1,036.1) | - |
| Choline (mg) | 292.5 (165.4) | 294.2 (164.6) | - |

N=25 participants. Number of participants taking supplements includes only targeted (named nutrient) products, and excludes multivitamins and complexes.

**Supplementary Table 2.** Nutrient and Dietary Quality Comparison with Migraine Attack Phase

| **Variable** | **Overall**  **(n=184)**  **Mean [CI]** | | **Interictal**  **(n=71)**  **Mean [CI]** | | **Prodrome (n=26)**  **Mean [CI]** | | **Migraine Pain (n=56)**  **Mean [CI]** | | **Postdrome (n=19)**  **Mean [CI]** | | **Other**  **Headache (n=11)**  **Mean [CI]** | |
| --- | --- | --- | --- | --- | --- | --- | --- | --- | --- | --- | --- | --- |
| Energy (kcal) | 1843 | 1739, 1947 | 1979 | 1784, 2175 | 1656 | 1481, 1830 | 1699 | 1560, 1838 | 1705 | 1422, 1989 | 2304 | 1750, 2858 |
| Fiber density | 11.6 | 10.6, 12.6 | 11.9 | 9.7, 14.1 | 11.5 | 9.5, 13.4 | 11.8 | 10.5, 13.2 | 9.4 | 7.6, 11.2 | 12.7 | 10.6, 14.7 |
| **% Calories From:** |  |  |  |  |  |  |  |  |  |  |  |  |
| Fat | 36.4 | 35.2, 37.5 | 36.2 | 34.7, 37.7 | 36.2 | 32.7, 39.7 | 36.7 | 34.6, 38.8 | 36.1 | 32.5, 39.7 | 35.6 | 30.7, 40.5 |
| Carbohydrate | 46.3 | 44.9, 47.6 | 46.9 | 44.9, 49.0 | 43.0 | **39.3, 46.7*** | 46.4 | 44.0, 48.8 | 46.6 | 42.1, 51.2 | 48.6 | 42.5, 54.7 |
| Protein | 16.2 | 15.4, 16.9 | 15.7 | 14.5, 16.9 | 19.6 | **17.1,22.0**** | 15.9 | 14.5, 17.3 | 15.1 | 13.5, 16.8 | 14.6 | 12.2, 17.1 |
| Saturated fat | 11.8 | 11.2, 12.4 | 12.1 | 11.2, 12.9 | 12.4 | 10.8, 14.1 | 11.5 | 10.5, 12.5 | 11.0 | 9.4, 12.6 | 11.2 | 8.8, 13.6 |
| MUFA | 12.9 | 12.4, 13.5 | 12.7 | 11.8, 13.6 | 12.7 | 10.9, 14.4 | 13.4 | 12.3, 14.5 | 13.5 | 11.9, 15.1 | 11.6 | 9.2, 13.9 |
| PUFA | 8.5 | 8.0, 9.0 | 8.3 | 7.6, 9.0 | 8.0 | 6.9, 9.0 | 8.7 | 7.8, 9.7 | 8.4 | 6.4, 10.5 | 9.5 | 7.2, 11.8 |
| Sugar | 17.9 | 16.8, 19.0 | 18.0 | 16.2, 19.7 | 17.3 | 14.5, 20.1 | 17.9 | 15.8, 20.1 | 18.7 | 14.5, 22.8 | 18.1 | 14.2, 22.0 |
| Animal protein | 9.9 | 9.1, 10.7 | 9.6 | 8.4, 10.8 | 13.4 | **10.8,16.1**** | 9.4 | 8.0, 10.9 | 9.4 | 7.5, 11.3 | 7.2 | 4.0, 10.4 |
| Plant protein | 6.4 | 6.1, 6.7 | 6.2 | 5.7, 6.7 | 6.1 | 5.1, 7.0 | 6.7 | 6.0, 7.3 | 5.9 | 5.0, 6.8 | 7.9 | **6.5, 9.3**** |
| Omega-3 | 0.9 | 0.9, 1.0 | 1.0 | 0.9, 1.1 | 1.0 | 0.8, 1.3 | 0.9 | 0.8, 1.0 | 0.8 | 0.6, 1.0 | 0.9 | 0.6, 1.1 |
| Omega-6 | 7.4 | 6.9, 7.9 | 7.1 | 6.4, 7.9 | 6.8 | 5.8, 7.7 | 7.7 | 6.9, 8.5 | 7.5 | 5.6, 9.4 | 8.5 | 6.1, 10.9 |
| Ultra-Processed | 64.4 | 66.7, 72.2 | 72.1 | 68.0, 76.4 | 64.7 | 57.6, 71.8 | 67.4 | 61.8, 73.0 | 74.0 | 65.4, 82.7 | 63.6 | 56.6, 70.6 |
| **HEI Components** |  |  |  |  |  |  |  |  |  |  |  |  |
| Total Score | 54.5 | 52.4, 56.7 | 54.1 | 50.6, 57.7 | 54.9 | 49.4, 60.4 | 54.8 | 51.2, 58.3 | 53.7 | 46.3, 61.1 | 59.0 | 50.7, 67.3 |
| Total Fruits | 2.1 | 1.9, 2.4 | 2.2 | 1.8, 2.7 | 2.8 | 2.0, 3.5 | 1.8 | 1.4, 2.3 | 1.7 | 0.8, 2.5 | 2.6 | 1.6, 3.6 |
| Whole Fruits | 2.7 | 2.4, 3.0 | 2.7 | 2.2, 3.2 | 3.5 | 2.6, 4.3 | 2.5 | 2.0, 3.1 | 2.2 | 1.2, 3.2 | 3.3 | 2.2, 4.4 |
| Total Vegetables | 3.0 | 2.8, 3.3 | 2.8 | 2.4, 3.2 | 3.2 | 2.6, 3.8 | 3.1 | 2.6, 3.5 | 2.9 | 2.2, 3.6 | 3.9 | **3.2, 4.7**** |
| Greens & Beans | 2.4 | 2.1, 2.7 | 2.3 | 1.8, 2.8 | 2.6 | 1.7, 3.4 | 2.4 | 1.8, 3.0 | 2.6 | 1.7, 3.6 | 2.8 | 1.3, 4.2 |
| Whole Grains | 4.0 | 3.4, 4.6 | 3.9 | 3.0, 4.9 | 3.0 | 1.6, 4.4 | 4.5 | 3.5, 5.5 | 4.1 | 2.3, 5.9 | 4.2 | 2.0, 6.5 |
| Dairy | 5.0 | 4.4, 5.5 | 5.5 | 4.6, 6.4 | 5.3 | 4.1, 6.6 | 4.3 | 3.3, 5.3 | 4.9 | 3.2, 6.7 | 4.0 | 2.6, 5.5 |
| Total Protein Foods | 4.1 | 3.9, 4.4 | 4.0 | 3.6, 4.4 | 4.6 | **4.4, 4.9**** | 4.1 | 3.7, 4.5 | 3.9 | 3.2, 4.6 | 4.5 | 4.0, 5.1 |
| Seafood & Plant Protein | 2.9 | 2.6, 3.2 | 2.8 | 2.2, 3.3 | 2.8 | 1.9, 3.7 | 3.0 | 2.4, 3.6 | 2.6 | 1.5, 3.7 | 3.8 | 2.7, 5.0 |
| Fatty Acid Ratio | 5.2 | 4.7, 5.7 | 4.9 | 4.1, 5.8 | 4.4 | 3.1, 5.8 | 5.7 | 4.7, 6.6 | 5.8 | 4.0, 7.5 | 5.5 | 3.2, 7.7 |
| Refined Grains | 5.4 | 4.9, 6.0 | 5.2 | 4.3, 6.1 | 5.8 | 4.3, 7.3 | 5.4 | 4.5, 6.4 | 5.7 | 4.1, 7.3 | 5.6 | 3.2, 8.1 |
| Sodium | 4.3 | 3.8, 4.9 | 4.2 | 3.4, 5.1 | 3.7 | 2.3, 5.2 | 4.6 | 3.6, 5.5 | 4.5 | 2.8, 6.2 | 5.3 | 3.0, 7.7 |
| Added Sugars | 8.0 | 7.6, 8.4 | 8.2 | 7.6, 8.8 | 8.2 | 7.8, 9.4 | 7.8 | 7.0, 8.7 | 7.0 | 5.5, 8.6 | 8.0 | 6.7, 9.3 |
| Saturated Fats | 5.4 | 4.9, 5.9 | 5.3 | 4.6, 6.1 | 4.9 | 3.6, 6.2 | 5.6 | 4.6, 6.5 | 5.7 | 4.1, 7.4 | 5.4 | 3.3, 7.5 |

** Difference from interictal p<0.05, adjusted and unadjusted model

* Difference from interictal p<0.05, adjusted model only

PUFA: Polyunsaturated Fatty Acids; MUFA: Monounsaturated Fatty Acids

|  | **Any Headache Pain (n=63)** | | | | | | **Only Migraine Pain (n=52)** | | | | | |
| --- | --- | --- | --- | --- | --- | --- | --- | --- | --- | --- | --- | --- |
| **Variable** | **Mild**  **(n=13)**  **Mean [CI]** | | **Moderate**  **(n=23)**  **Mean [CI]** | | **Severe**  **(n=27)**  **Mean [CI]** | | **Mild**  **(n=10)**  **Mean [CI]** | | **Moderate (n=18)**  **Mean [CI]** | | **Severe**  **(n=24)**  **Mean [CI]** | |
| Energy (kcal) | 1684 | 1459, 1909 | 2006 | 1749, 2263 | 1684 | 1407, 1962 | 1537 | 1322, 1751 | 1849 | 1620, 2079 | 1646 | 1415, 1877 |
| Fiber density | **15.6*** | **12.8, 18.3** | 10.5 | 9.2, 12.0 | 11.1 | 9.2, 13.0 | **15.4*** | **12.0, 18.8** | 10.2 | 8.5, 11.9 | 11.2 | 9.1, 13.2 |
| **% Calories From:** |  |  |  |  |  |  |  |  |  |  |  |  |
| Total Fat | 39.9 | 36.0, 43.9 | 35.6 | 31.8, 39.3 | 36.8 | 34.0, 39.5 | 41.1 | 36.8,45.5 | 36.7 | 32.5, 41.0 | 36.1 | 33.2, 38.9 |
| Carbohydrate | 43.8 | 39.4, 48.2 | 47.9 | 43.7, 52.1 | 45.8 | 42.6, 49.0 | 42.4 | 37.3, 47.5 | 46.2 | 41.6, 50.7 | 46.6 | 43.4, 49.8 |
| Protein | 16.2 | 14.0,18.5 | 15.9 | 13.8, 18.0 | 15.7 | 13.8, 17.6 | 16.4 | 13.5, 19.3 | 16.9 | 14.6, 19.3 | 15.4 | 13.4, 17.5 |
| Saturated fat | 12.3 | 10.8, 13.7 | 11.0 | 9.4, 12.6 | 11.8 | 10.2, 13.4 | 12.1 | 10.6, 13.5 | 11.4 | 9.7, 13.2 | 11.7 | 9.9, 13.5 |
| MUFA | 14.4 | 12.2, 16.7 | 12.6 | 11.0, 14.2 | 13.5 | 12.0, 15.1 | 15.3 | 13.0, 17.5 | 13.2 | 11.5, 14.9 | 13.5 | 11.9, 15.2 |
| PUFA | 10.0 | 7.7, 12.4 | 8.9 | 7.6, 10.2 | 8.3 | 7.1, 9.4 | **10.6*** | **7.9, 13.3** | 9.0 | 7.4, 10.6 | 7.8 | 6.8, 8.7 |
| Sugar | 15.1 | 12.0, 18.1 | 19.2 | 15.2, 23.1 | 17.8 | 15.1, 20.5 | 14.6 | 10.8, 18.5 | 18.5 | 13.9, 23.1 | 18.3 | 15.4, 21.3 |
| Animal protein | 8.7 | 6.2, 11.3 | 9.9 | 7.4, 12.3 | 9.1 | 7.2, 11.1 | 8.8 | 5.5, 12.1 | 11.4 | 8.8, 14.0 | 8.9 | 7.0, 10.9 |
| Plant protein | 8.0 | 6.9, 9.1 | 6.2 | 5.3, 7.2 | 6.8 | 5.8, 7.8 | 8.0 | 6.7, 9.4 | 5.6 | 4.8, 6.5 | 6.7 | 5.7, 7.7 |
| Omega-3 | 1.0 | 0.7, 1.4 | 0.9 | 0.7, 1.1 | 0.8 | 0.6, 0.9 | **1.2*** | **0.7, 1.6** | 1.0 | 0.7, 1.2 | 0.7 | 0.6, 0.8 |
| Omega-6 | 8.9 | 6.8, 10.9 | 7.8 | 6.6, 9.0 | 7.4 | 6.2, 8.5 | **9.3*** | **7.0, 11.6** | 7.8 | 6.3, 9.3 | 6.9 | 6.1, 7.8 |
| Ultra-processed | 63.1 | 49.3, 76.9 | 69.5 | 61.9, 77.1 | 65.3 | 58.4, 72.2 | 63.4 | 46.7, 80.1 | 70.9 | 61.6, 80.2 | 65.5 | 57.8, 73.2 |
| **HEI Components** |  |  |  |  |  |  |  |  |  |  |  |  |
| Total Score | **61.9*** | **53.8, 69.9** | 54.6 | 49.8, 59.5 | 52.6 | 47.5, 57.8 | 61.0 | 53.6, 68.4 | 54.1 | 48.5, 59.7 | 52.1 | 46.3, 57.8 |
| Total Fruits | 2.3 | 1.4, 3.2 | 1.9 | 1.2, 2.6 | 1.9 | 1.2, 2.6 | 2.3 | 1.2, 3.4 | 1.6 | 0.9, 2.4 | 1.8 | 1.1, 2.5 |
| Whole Fruits | 3.2 | 2.1, 4.3 | 2.7 | 1.8, 3.6 | 2.4 | 1.6, 3.2 | 3.2 | 1.8, 4.5 | 2.4 | 1.5, 3.4 | 2.3 | 1.4, 3.2 |
| Total Vegetables | **4.4*** | **3.9, 5.0** | 2.7 | 2.1, 3.4 | 3.1 | 2.5, 3.8 | **4.3*** | **3.6, 5.0** | 2.5 | 1.7, 3.2 | 3.1 | 2.4, 3.8 |
| Greens & Beans | **3.8*** | **2.7, 4.9** | 2.1 | 1.2, 3.0 | 2.3 | 1.4, 3.1 | 3.9 | 2.6, 5.1 | 1.8 | 0.9, 2.7 | 2.4 | 1.5, 3.2 |
| Whole Grains | 4.4 | 2.5, 6.3 | 3.9 | 2.3, 5.6 | 4.3 | 2.9, 5.7 | 3.0 | 1.3, 4.6 | 4.6 | 2.7, 6.5 | 4.4 | 2.9, 5.9 |
| Dairy | 4.3 | 2.4, 6.1 | 4.2 | 2.8, 5.6 | 4.2 | 2.8, 5.6 | 4.0 | 1.8, 6.3 | 4.2 | 2.5, 6.0 | 4.3 | 2.9, 5.8 |
| Total Protein Foods | 4.7 | 4.3, 5.1 | 4.2 | 3.6, 4.7 | 4.1 | 3.5, 4.7 | 4.9 | 4.6, 5.1 | 4.1 | 3.4, 4.8 | 4.0 | 3.4, 4.7 |
| Seafood & Plant Protein | 3.7 | 2.6, 4.8 | 3.1 | 2.2, 4.0 | 2.9 | 2.0, 3.8 | 4.1 | 2.9, 5.2 | 2.9 | 1.9, 3.9 | 2.6 | 1.6, 3.6 |
| Fatty Acid Ratio | 6.7 | 4.8, 8.5 | 5.8 | 4.3, 7.3 | 5.1 | 3.7, 6.5 | 7.1 | 5.2, 9.1 | 5.8 | 4.1, 7.4 | 5.1 | 3.6, 6.6 |
| Refined Grains | 5.9 | 3.7, 8.1 | 5.5 | 3.9, 7.0 | 5.3 | 4.0, 6.5 | 5.6 | 3.2, 8.0 | 5.6 | 3.9, 7.4 | 5.2 | 4.0, 6.5 |
| Sodium | 4.6 | 2.3, 6.8 | 5.0 | 3.6, 6.3 | 4.2 | 2.9, 5.6 | 4.3 | 1.6, 6.9 | 5.0 | 3.7, 6.4 | 4.0 | 2.7, 5.4 |
| Added Sugars | 9.2 | 8.7, 9.8 | 7.5 | 6.2, 8.8 | 7.8 | 6.5, 9.0 | 9.5 | 9.0, 10.0 | 7.6 | 6.1, 9.1 | 7.6 | 6.2, 8.9 |
| Saturated Fats | 4.8 | 3.1, 6.5 | 6.1 | 4.7, 7.5 | 5.1 | 3.7, 6.6 | 4.9 | 3.1, 6.7 | 5.9 | 4.3, 7.5 | 5.2 | 3.7, 6.8 |

**Supplementary Table 3.** Nutrient and Dietary Quality Comparison with Pain Intensity

*p<0.05 compared to severe pain reference.

PUFA: Polyunsaturated Fatty Acids; MUFA: Monounsaturated Fatty Acids

**Supplementary Figure 1**: Study Design Flow Diagram


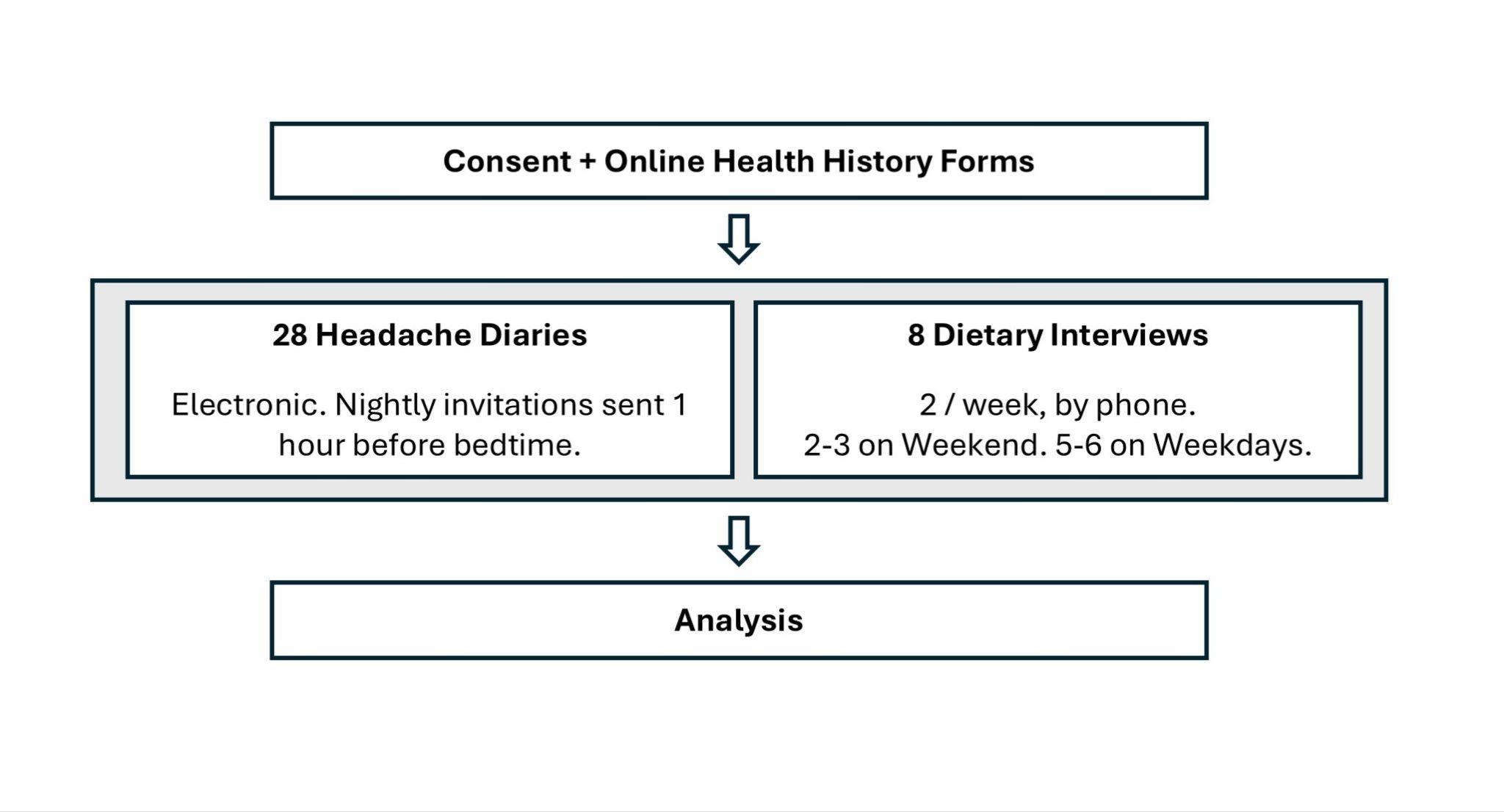


**Supplementary Figure 2**: Participants Included and Excluded


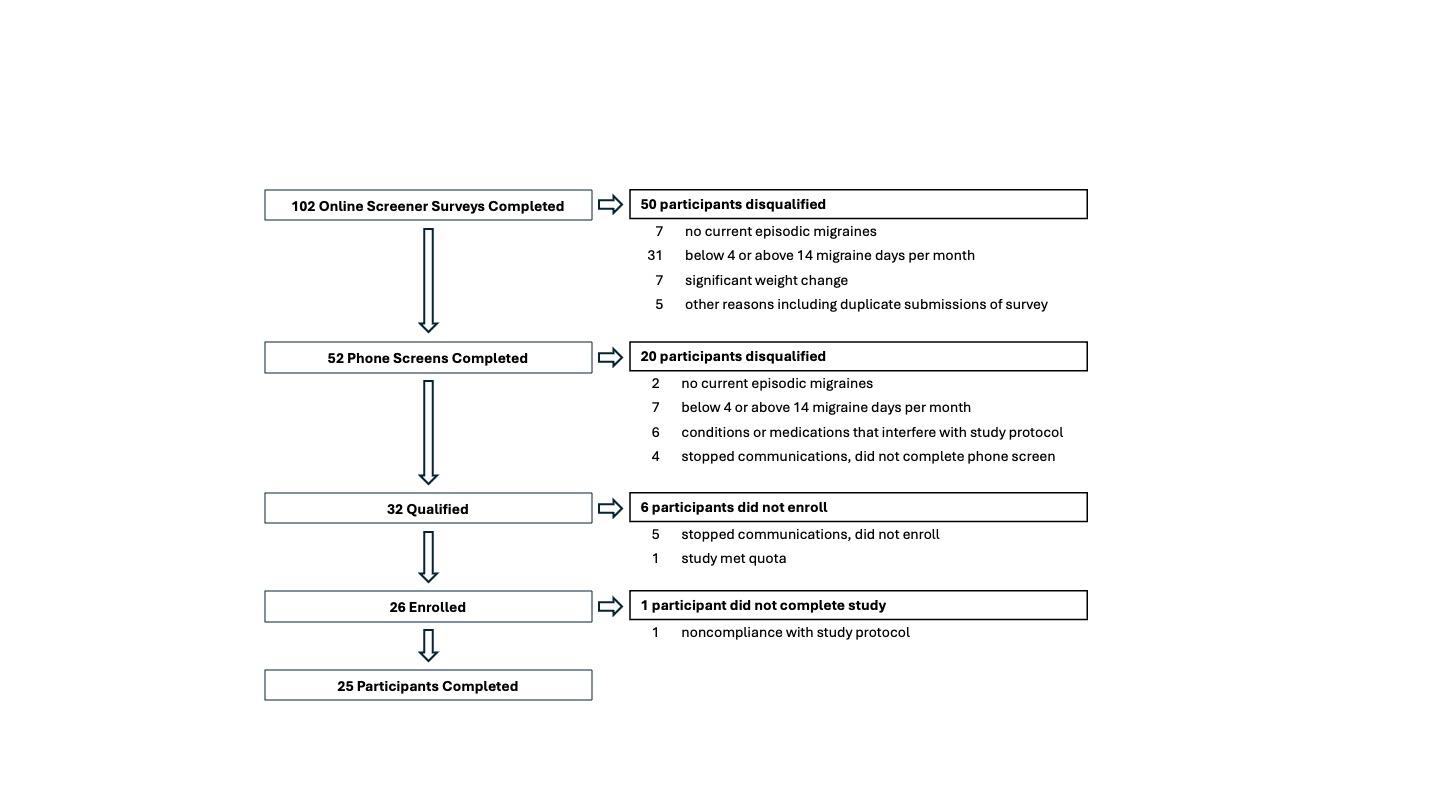

Supplement: Multimedia component 1 [file mmc1.docx]
